# High-Precision and Lightweight Model for Rapid Safety Helmet Detection

**DOI:** 10.3390/s24216985

**Published:** 2024-10-30

**Authors:** Xuejun Jia, Xiaoxiong Zhou, Chunyi Su, Zhihan Shi, Xiaodong Lv, Chao Lu, Guangming Zhang

**Affiliations:** 1 College of Electrical Engineering and Control Science, Nanjing Tech University, Nanjing 211816, China; jxj@njtech.edu.cn (X.J.); zhouxx@njtech.edu.cn (X.Z.); chunysu@163.com (C.S.); szh@njtech.edu.cn (Z.S.); lvlvxiaodong@126.com (X.L.); 202361206143@njtech.edu.cn (C.L.); 2 China Construction Second Engineering Bureau Co., Ltd., Beijing 100160, China

**Keywords:** YOLOv5s, CBAM attention mechanism, MPDIoU loss function, safety helmet detection

## Abstract

This paper presents significant improvements in the accuracy and computational efficiency of safety helmet detection within industrial environments through the optimization of the you only look once version 5 small (YOLOv5s) model structure and the enhancement of its loss function. We introduce the convolutional block attention module (CBAM) to bolster the model’s sensitivity to key features, thereby enhancing detection accuracy. To address potential performance degradation issues associated with the complete intersection over union (CIoU) loss function in the original model, we implement the modified penalty-decay intersection over union (MPDIoU) loss function to achieve more stable and precise bounding box regression. Furthermore, considering the original YOLOv5s model’s large parameter count, we adopt a lightweight design using the MobileNetV3 architecture and replace the original squeeze-and-excitation (SE) attention mechanism with CBAM, significantly reducing computational complexity. These improvements reduce the model’s parameters from 15.7 GFLOPs to 5.7 GFLOPs while increasing the mean average precision (mAP) from 82.34% to 91.56%, demonstrating its superior performance and potential value in practical industrial applications.

## 1. Introduction

The safety helmet is a crucial piece of protective equipment on construction sites, capable of protecting workers from head injuries caused by falling objects and collisions. Wearing a safety helmet is an important measure to ensure workers’ safety, effectively reducing the risk of head injuries. However, despite the clear importance of safety helmets, many workers are often reluctant to wear them during construction for various reasons. This reluctance to wear helmets poses significant safety hazards, highlighting the urgent need for effective monitoring of safety helmet usage on construction sites. Traditional monitoring methods primarily rely on manual inspection, which is not only resource-intensive but also inefficient, prone to errors, and management oversights. Given these challenges, automated monitoring systems that incorporate sensor technology have become an important research direction.

In the application of sensor technology, cameras, as a key sensor, can capture and analyze site images in real-time to determine whether workers are wearing safety helmets. Compared to traditional manual inspection methods, camera-based monitoring systems offer advantages such as automation, high accuracy, and strong real-time capabilities. However, the large and complex data collected by cameras presents the challenge of quickly and accurately identifying instances of workers not wearing safety helmets within the vast amount of images, which has become a focal point of research in this field. Therefore, the combination of advanced image recognition algorithms with computationally lightweight models is particularly important. This article presents an effective and streamlined approach to detecting safety helmets using a refined YOLOv5 algorithm. This approach integrates attention mechanisms and optimizes the model to boost detection precision and enhance processing speed, thereby providing robust support for tracking safety helmet compliance at construction sites. These algorithms sustain high accuracy while minimizing the use of computational resources, rendering the detection system more viable and efficient.

Recent progress in machine learning has facilitated the creation of more accurate and efficient safety helmet detection systems, greatly diminishing the labor expenses linked to manual monitoring. For example, the fast object detection algorithm based on a cascade classifier, proposed by Viola and Jones, greatly improved detection speed and accuracy [1]. Dalal and Triggs’ histogram of oriented gradients (HOG) method enhances feature extraction efficiency by analyzing the distribution of gradient directions in images [2]. Moreover, Felzenszwalb et al.’s deformable part models (DPM) added robustness to detection models by flexibly representing object parts [3]. These foundational methods laid the groundwork for advancements in object detection.

The emergence of deep learning, especially convolutional neural networks (CNNs), has brought significant breakthroughs in object detection. Redmon et al. introduced the you only look once (YOLO) algorithm, which reconceptualized object detection as a regression problem, facilitating real-time detection [4]. Girshick et al.’s Fast R-CNN algorithm improved detection efficiency and accuracy through region proposal networks (RPNs) [5]. He et al. expanded the scope of detection models with the Mask R-CNN algorithm, which adds instance segmentation capabilities [6].

Beyond traditional CNNs, recent deep learning models have excelled in processing large-scale data and complex environments. Lin et al.’s focal loss algorithm improved the performance of single-stage detection models by weighting difficult-to-detect samples more heavily [7]. Tan and Le’s EfficientDet algorithm, which combines the EfficientNet backbone with the BiFPN feature pyramid, achieved higher accuracy and speed in detection tasks [8]. These models have made substantial strides in enhancing detection efficiency and precision.

To further improve model performance, object detection models have incorporated attention mechanisms to enhance the model’s capacity for representation and extract more relevant features. For instance, Hu et al.’s squeeze-and-excitation network (SENet) increased the model’s feature representation capability by recalibrating inter-channel relationships [9]. Woo et al. developed the convolutional block attention module (CBAM), which integrates spatial and channel attention mechanisms to further enhance model performance [10]. These attention mechanisms have found widespread application in object detection, natural language processing, and other domains.

Although many newer versions with superior performance (such as YOLOv6, YOLOv7, and YOLOv8) have emerged following YOLOv5, we chose YOLOv5s as the basis of our study for several reasons. First, YOLOv5s is widely adopted in practical industrial applications, particularly performing well on resource-constrained devices such as embedded systems and mobile devices. Second, YOLOv5s’s lightweight design and relatively low computational cost make it more suitable for real-time detection tasks. In contrast, newer versions, although superior in certain scenarios, often come with higher computational demands, which may not be ideal for real-time applications in industrial environments. Therefore, our study is based on YOLOv5s, and we further optimize its model structure and loss function to improve detection performance, ensuring efficient object detection even under resource-limited conditions.

This research focuses on the in-depth exploration and application of attention mechanisms, alongside systematic enhancements to the YOLOv5s algorithm across multiple stages and layers. The goal of these improvements is to enable the model to extract features more effectively and handle complex data, leading to significant advancements in detection accuracy, stability, and generalization. The main contributions of this paper to the YOLOv5s model are threefold:

Incorporation of the CBAM attention mechanism: The convolutional block attention module (CBAM) is an efficient attention mechanism designed to improve the network’s ability to extract relevant features by concentrating on critical parts of the image. In the context of safety helmet detection, CBAM helps the model focus on key details, such as the positions of the head and helmet, thereby enhancing the accuracy of safety helmet recognition.

Enhancement of the loss function with MPDIoU: The conventional CIoU loss function may be inadequate in complex scenarios, particularly when the target objects are highly overlapping or severely occluded. To overcome this limitation, this study introduces the modified penalized distance intersection over union (MPDIoU) loss function, which refines the bounding box regression strategy, thus improving the model’s stability and accuracy in challenging situations.

Model structure optimization using MobileNetV3: Given the substantial parameter count and computational complexity of the YOLOv5s model, this study integrates MobileNetV3 as the backbone network, substituting the original SE attention mechanism with CBAM. These modifications considerably lower the model’s parameters and computational demands, rendering it more apt for deployment on devices with limited resources and broadening its practical use in industrial environments.

By thoroughly investigating and refining these technical aspects, this study not only enhances the performance of safety helmet detection but also offers new insights and methodologies for future research in this domain. Furthermore, the paper explores the potential challenges these improvements may introduce and discusses future directions for development, with the aim of providing a more efficient and precise safety helmet detection solution for industrial, construction, and manufacturing environments. This approach is expected to better ensure employee safety and reduce the incidence of workplace accidents. Additionally, the research aspires to stimulate further technological innovation and application exploration, advancing progress in safety-related fields.

This paper is organized into the following three sections for detailed discussion: Section 2 introduces the YOLOv5s algorithm and summarizes its core principles and architecture. Section 3 outlines the proposed modifications, such as incorporating the CBAM) to improve detection accuracy, adopting the MPDIoU loss function to resolve the shortcomings of the CIoU, and replacing the YOLOv5s backbone with the MobileNetV3 architecture to decrease the model’s parameter size. Section 4 describes the experimental condition, including dataset collection and model training outcomes. This section details the experimental configuration, covering dataset preprocessing, model training parameters, and technical aspects. A comparative analysis of the results is presented, highlighting key improvements in detection accuracy and efficiency. Finally, Section 5 concludes the paper by summarizing the research, reviewing major contributions and findings, and outlining the model’s practical applications, challenges, and future research directions.

## 2. YOLOv5s Algorithm

YOLOv5 was developed by the Ultralytics team. Although it is not an official continuation of the original YOLO by its creators, its excellent performance and ease of use have made it widely adopted in the field of object detection [11]. YOLOv5 provides various model variants, enabling it to meet the demands of different computational resource conditions while maintaining real-time detection performance [12].

The network structure of YOLOv5 consists of three main components: the backbone network (backbone), the neck (neck), and the head (head). The backbone network uses CSPNet (cross-stage partial network), a structure that enhances the feature representation capability and computational efficiency of the network through cross-stage partial connections [13]. The neck structure employs PANet (path aggregation network) to fuse multi-scale features, thereby improving detection accuracy [14]. The head is responsible for the final bounding box regression and class prediction.

**Figure 1:**
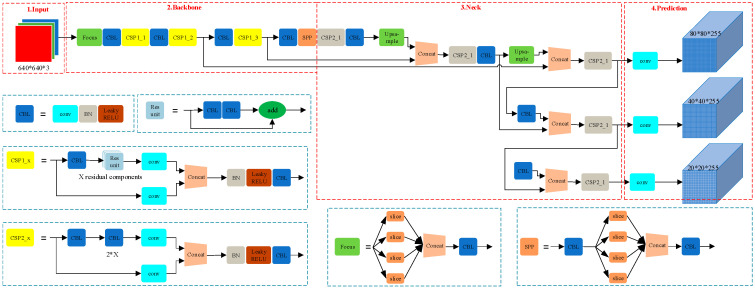
YOLOv5 network structure.

As illustrated in Figure 1, the backbone network of YOLOv5 extracts deep image features through multi-level convolution and pooling operations. The PANet structure in the neck aggregates features from different scales to capture detailed information on objects of various sizes. The head then produces the final detection outputs, including object classes and locations.

YOLOv5 provides several model variants to cater to different computational resource and accuracy needs, including YOLOv5s, YOLOv5m, YOLOv5l, and YOLOv5x. These variants vary in terms of model parameters and computational complexity [15]:

YOLOv5s: The smallest variant, ideal for resource-limited devices, offering high processing speed but lower accuracy.

YOLOv5m: A mid-sized variant, balancing parameters, computation, and accuracy.

YOLOv5l: A larger variant, suited for applications requiring higher accuracy.

YOLOv5x: The largest variant, providing the highest accuracy, but with the greatest computational demand.

YOLOv5 Loss Function

The YOLOv5 loss function is composed of three main components: Localization loss, confidence loss, and class loss. In the localization loss component, YOLOv5 employs the CIoU loss function. CIoU not only considers the overlap area between bounding boxes but also integrates the distance between their center points and aspect ratio, resulting in more accurate bounding box regression [16].

(1) $$L_{CIoU}=1-IoU+\frac{\rho^{2}(b,b^{gt})}{c^{2}}+\alpha v$$

In this formula, IoU represent Intersection over Union, $\rho^{2}(b,b^{gt})$ is the Euclidean distance between the center points of the ground truth box and the predicted box, c represents the diagonal length of the smallest enclosing box that includes both the predicted and ground truth boxes, α is a weighting factor for aspect ratio consistency, and v assesses the consistency of aspect ratios between the predicted boxes and the ground truth.

Additionally, the confidence loss evaluates how well the predicted box overlaps with the ground truth box, while the class loss assesses the accuracy of object classification. The overall loss function for YOLOv5 is expressed as:

(2) $$L=L_{CIoU}+L_{conf}+L_{cls}$$

where $L_{CIoU}$ denotes the CIoU loss, $L_{conf}$ represents the confidence loss, and $L_{cls}$ indicates the class loss.

Formulas (1) and (2) are the standard loss function expressions in YOLOv5, which include three parts: CIoU loss, confidence loss, and category loss. Although these formulas are not new, they are key components of the YOLOv5 algorithm. In our work, we use these basic loss functions and optimize them. For example, to address the problem of inaccurate positioning of CIoU when dealing with overlapping objects, we introduced the modified penalty-decay IoU (MPDIoU) loss function to further improve the positioning accuracy. In addition, we improved the network structure by integrating the lightweight MobileNetV3 architecture and the CBAM attention mechanism, effectively reducing the computational complexity while maintaining high-precision detection.

Thanks to its efficient architecture, diverse model variants, and integrated loss function design, YOLOv5 excels in object detection tasks. Its ability to fuse multi-scale features and extract them efficiently ensures high accuracy and performance even in challenging scenarios.

## 3. Improved YOLOv5s Algorithm

While YOLOv5 is a highly efficient algorithm in object detection, excelling in both speed and accuracy, it has certain limitations. For instance, in complex scenes, its detection accuracy can diminish, especially when handling small or densely packed objects. Moreover, the CIoU loss function used in YOLOv5 may not always yield the optimal bounding box regression, potentially compromising detection performance.

### 3.1. Introduction of the CBAM Attention Mechanism

Attention modules have recently been widely adopted to improve object detection performance [17,18,19,20]. The core concept of attention mechanisms is to increase model accuracy by directing focus to key features within images and understanding their interrelationships. Initially applied in natural language processing, attention mechanisms have been increasingly utilized in computer vision tasks due to their notable effectiveness in image processing.

**Figure 2:**
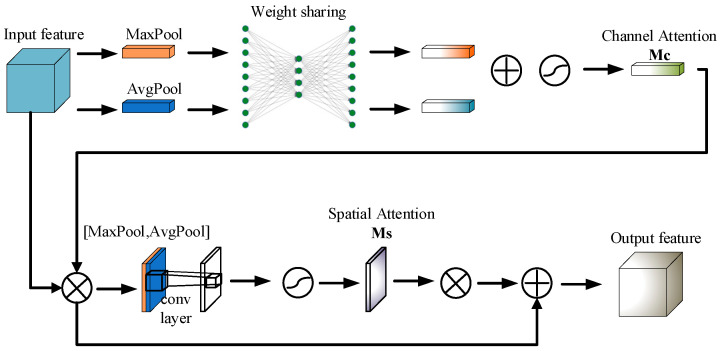
CBAM module structure.

The CBAM integrates both channel and spatial attention mechanisms, significantly boosting the effectiveness of CNN [21]. CBAM consists of two essential parts: channel attention and spatial attention. The channel attention mechanism employs fully connected layers, pooling layers, and the sigmoid activation function to determine the importance weights for each channel. First, the input feature map undergoes global average pooling and global max pooling to capture global contextual information. The pooled outputs proceed through shared, fully connected layers and activation functions to produce attention weights for individual channels. These weights are then applied to the input feature map through element-wise multiplication, accentuating the most relevant channels. CBAM module structure shown as Figure 2.

Mathematically, the channel attention mechanism can be represented as:

(3) $$M_{c}(F)=\sigma (W_{1}(W_{0}(F_{avg}))+W_{1}(W_{0}(F_{max})))$$

Here, $F_{avg}$ and $F_{max}$ represent the results of global average pooling and global max pooling, respectively, $W_{0}$ and $W_{1}$ are the shared fully connected layers, and σ is the sigmoid activation function.

Mathematically, the spatial attention mechanism can be expressed as:

(4) $$M_{s}(F)=\sigma (f^{7\times 7}([F_{avg};F_{max}]))$$

Here, $F_{avg}$ and $F_{max}$ represent the results of average pooling and max pooling across the channel dimension, $f^{7\times 7}$ denotes the 7 × 7 convolution operation, and σ is the sigmoid activation function.

### 3.2. MPDIoU Loss

To enhance helmet detection accuracy, this study introduces MPDIoU, a precise bounding box regression (BBR) loss function that measures similarity using the minimum point distance intersection over union [22]. It extends the traditional IoU concept, refining how the overlapping region between predicted and actual boxes is determined. This approach particularly resolves the limitations of GIoU when there is significant overlap between the two boxes. IoU measures the proportion of the intersection area to the union area of the predicted and actual boxes, as described by the following formula:

(5) $$IoU=\frac{A\cap B}{A\cup B}$$

**Figure 3:**
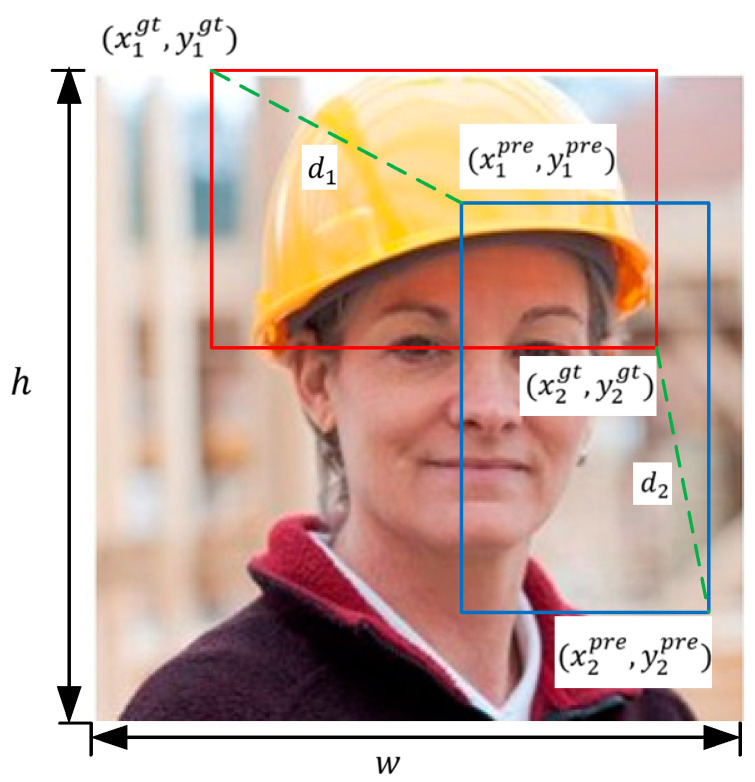
MPDIoU schematic diagram.

In object detection tasks, we define the ground truth box as A and the predicted box as B. A∩B denotes the area of the intersection between the two boxes, while A∪B represents the area of their union. First, we obtain the coordinates of the top-left corner $\left(x_{1}^{pre},\;y_{1}^{pre}\right)$ and the bottom-right corner $\left(x_{2}^{pre},\;y_{2}^{pre}\right)$ of the predicted box, along with the corresponding coordinates $\left(x_{1}^{gt},\;y_{1}^{gt}\right)$ and $\left(x_{2}^{gt},\;y_{2}^{gt}\right)$ of the ground truth box. These coordinates, together with the feature map’s width w and height h, are used. The diagram shown Figure 3.

The improved MPDIoU can be calculated as follows, combining the concepts of IoU and point distance to enhance the precision of box localization.

(6) $$d_{1}^{2}=(x_{1}^{pre}-x_{1}^{gt}{)}^{2}+(y_{1}^{pre}-y_{1}^{gt}{)}^{2}$$

(7) $$d_{2}^{2}=(x_{2}^{pre}-x_{2}^{gt}{)}^{2}+(y_{2}^{pre}-y_{2}^{gt}{)}^{2}$$

(8) $$MPDIoU=\frac{A\cap B}{A\cup B}-\frac{d_{1}^{2}}{w^{2}+h^{2}}-\frac{d_{2}^{2}}{w^{2}+h^{2}}$$

(9) $$L_{MPDIoU}=1-MPDIoU$$

where $d_{1}^{2}$ is the square of the vertex distance between the predicted box and the upper left corner of the real box, $d_{2}^{2}$ is the square of the distance to the lower right vertex.

### 3.3. Improved MobileNetV3 as the Backbone Network

MobileNetV3, optimized using neural architecture search (NAS), builds on MobileNetV1 and MobileNetV2 by retaining depthwise separable convolutions and linear bottleneck residuals [23]. Besides these elements, MobileNetV3 incorporates the squeeze-and-excitation (SE) module, improving the model’s capacity to highlight important features. To reduce the computational cost of the Swish activation function, h-swish is employed as an efficient approximation, ensuring the model remains suitable for resource-constrained platforms like mobile devices while maintaining accuracy.

By applying a nonlinear transformation to the product of the input x and the sigmoid function, the model enhances positive values while suppressing negative ones. The inclusion of SE helps the model concentrate on critical information when processing complex visual tasks, thereby boosting overall performance and efficiency.

(10) Swish x=x·δ(x)

(11) $$h-wish\;x=x\cdot [\frac{ReLU(6(x+3))}{6}]$$

**Figure 4:**
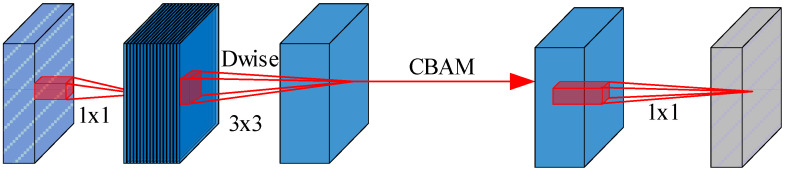
Improved MobileNetV3 network structure.

By applying a nonlinear transformation to the product of the input x and the sigmoid function, the model enhances the amplification of positive values while suppressing negative ones. The introduction of SE allows the model to concentrate more on critical information when processing complex visual tasks, thereby improving overall performance and efficiency. The structure shown as Figure 4.

When integrating MobileNetV3 as the feature extraction framework for YOLOv5, we observed that although using MobileNetV3 instead of the traditional Darknet53 significantly reduces model complexity, it has a tendency to miss pedestrians who are either far away or small in size. This issue mainly stems from the original squeeze-and-excitation (SE) module, which emphasizes inter-channel correlations but overlooks the significance of spatial features. Research indicates that spatial attention modules are more effective at identifying distant and small-scale objects. Consequently, we introduced the CBAM module to replace the SE module within MobileNetV3.

By incorporating the improved MobileNetV3 into YOLOv5, our model has significantly enhanced its capability to detect targets of varying sizes and distances in complex environments, all while maintaining low complexity. This improvement is particularly evident in urban traffic scenarios and densely populated areas. This advancement not only increases the model’s practicality but also opens up possibilities for real-time applications in resource-constrained settings.

## 4. Experiments and Analysis

### 4.1. Experimental Environment and Dataset

The Experimental environment shown as Table 1.

**Figure 5:**
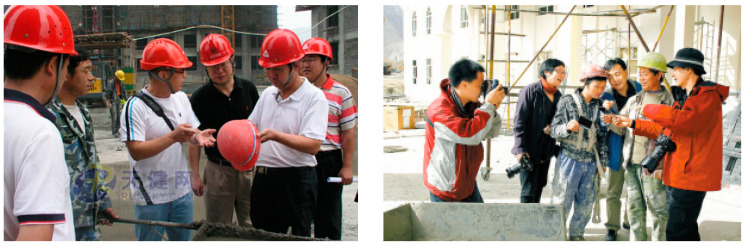
Training samples.

This study tests the effectiveness of the helmet detection algorithm using the open-source “Safety Helmet Wearing Dataset” (SHWD). SHWD consists of 7581 images, capturing various environments, lighting conditions, viewing angles, personal postures, and occlusion levels. The dataset comprises 9044 positive samples (helmet wearing) and 111,514 negative samples (non-helmet). It is partitioned into training and validation sets with a ratio of 80:20, using 6064 images for training and 1517 for validation. Figure 4 presents sample images from the training set. The example training samples shown as Figure 5.

Training of the model occurred in two stages: frozen and unfrozen training. The process was carried out over 100 epochs in total. During the first 50 epochs, frozen training was performed, keeping the backbone network parameters fixed. In the next 50 epochs, unfrozen training allowed the backbone parameters to be updated. The image resolution was set to 640 × 640, with a batch size of 32, an initial learning rate of 0.001, and an IoU threshold of 0.5. Model parameters were optimized using the Adam optimizer. 

### 4.2. Evaluation Metrics 

In this study, two evaluation metrics were used: mean average precision (mAP) and FLOPs.

The effectiveness of the helmet detection model is assessed by how accurately it identifies helmet usage. Correct detections indicate successful identification of individuals wearing helmets, while incorrect detections occur when the model mistakenly identifies someone as wearing a helmet.

True negatives indicate when the model correctly identifies individuals not wearing helmets, and false negatives are instances where the model misses detecting a helmet on someone who is actually wearing one.

A critical metric for evaluating the model is average precision (AP), which measures the average accuracy of the model across all possible recall rates, reflecting the model’s capability to recognize specific categories. The formula for calculating AP is:

(12) $$AP=\frac{TP+TN}{TP+TN+FP}$$

The mean average precision (mAP) provides a more comprehensive assessment, as it averages the AP values across all categories, making it a common metric for evaluating detection accuracy in object detection tasks. It is calculated by summing the AP values of all target classes and dividing the result by the total number of classes. The formula is as follows:

(13) $$mAP=\frac{\sum AP}{N}$$

Here, N represents the number of target categories.

These metrics allow us to quantify the model’s performance in detecting whether a helmet is being worn, enabling us to optimize model parameters and enhance detection accuracy.

Floating point operations (FLOPs) measure a model’s computational complexity, showing the number of operations needed for one forward pass. It helps assess the resource usage and computational load, especially in resource-limited environments. The formula for calculating FLOPs is provided below:

(14) $$\mathrm{FLOPs}=2\times H\times W\times (C_{in}\times K\times K)\times C_{out}$$

In this formula, H and W represent the height and width of the input to the convolution operation, $C_{in}$ is input channels, K is the size of the convolution kernel, and $C_{out}$ is output channels. The formula illustrates the number of multiply–accumulate operations required for each convolution, where the “2” accounts for the fact that each convolution involves one multiplication and one addition.

By calculating a model’s FLOPs, we can quantify its computational load during task execution, allowing us to assess its efficiency and suitability. This is especially important when designing models that need to be both efficient and practical.

### 4.3. Results and Analysis

A series of ablation experiments were conducted to verify the feasibility and effectiveness of the proposed improvements. These included: ① YOLOV5s, ② YOLOV5s+SE, ③ YOLOV5s+CBAM, ④ YOLOV5s+CBAM+MPDIoU, ⑤ YOLOV5s+MobileNetV3+MPDIoU, and ⑥ improved YOLOV5s (YOLOV5s+MobileNetV3+CBAM+MPDIoU). The detailed experimental results are presented in Table 2:

The results indicate that precision and recall are critical metrics for evaluating model performance. The improved YOLOv5s achieved the highest precision (93.81%) and recall (86.33%), demonstrating its superior performance in target localization and identification. In contrast, the baseline model (Model 1) showed lower precision and recall, at 89.56% and 75.41%, respectively. This substantial improvement suggests that integrating MobileNetV3 with CBAM and MPDIoU effectively enhances the model’s recognition capabilities.

mAP@0.5 is another vital performance metric used to assess the detector’s performance across various confidence thresholds. On this metric, improved YOLOv5s also excels, achieving 91.56%, indicating stable detection accuracy across diverse scenarios. Model 4 (YOLOv5s+CBAM+MPDIoU) also demonstrated a high mAP@0.5 of 87.70%, showing that the inclusion of CBAM and MPDIoU significantly boosts detection performance.

Regarding computational cost, the FLOPs for Model 5 and improved YOLOv5s were significantly reduced to 5.7G, compared to 15.8G for other models. This substantial reduction highlights that incorporating MobileNetV3 effectively lowers the computational burden, which is crucial for deployment in resource-limited environments.

**Figure 6:**
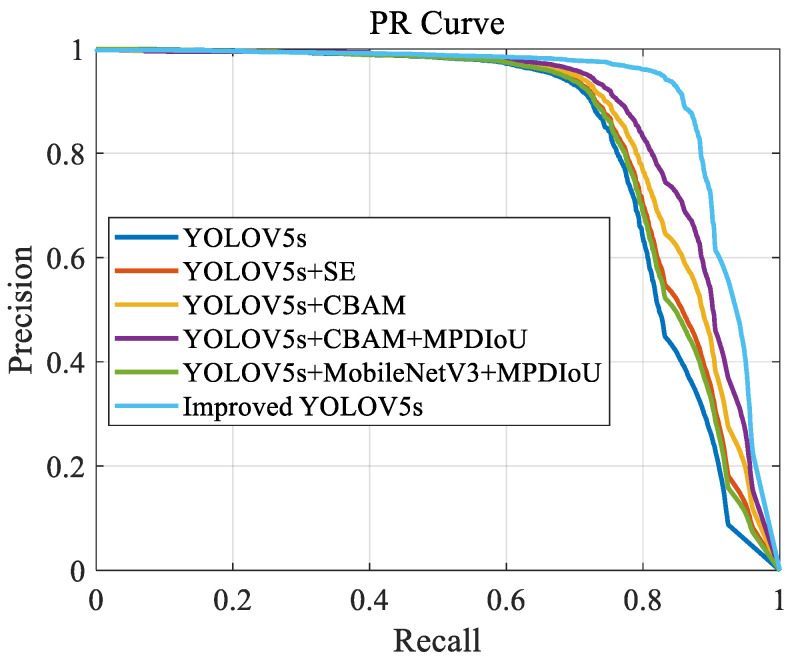
PR curve.

The PR curves illustrating the models’ performance are plotted as Figure 6:

The provided PR curve illustrates notable performance differences among the models. The improved YOLOv5s demonstrates the best performance, with its PR curve consistently positioned at the top, indicating superior precision across the entire recall range. This suggests that integrating MobileNetV3, CBAM, and MPDIoU technologies effectively enhances the accuracy and robustness of object detection in the improved YOLOv5s. Other models, such as YOLOv5s+CBAM and YOLOv5s+CBAM+MPDIoU, also show high precision, especially in the mid-to-high recall range, indicating that their improvement strategies significantly bolster their ability to handle complex scenarios.

**Figure 7:**
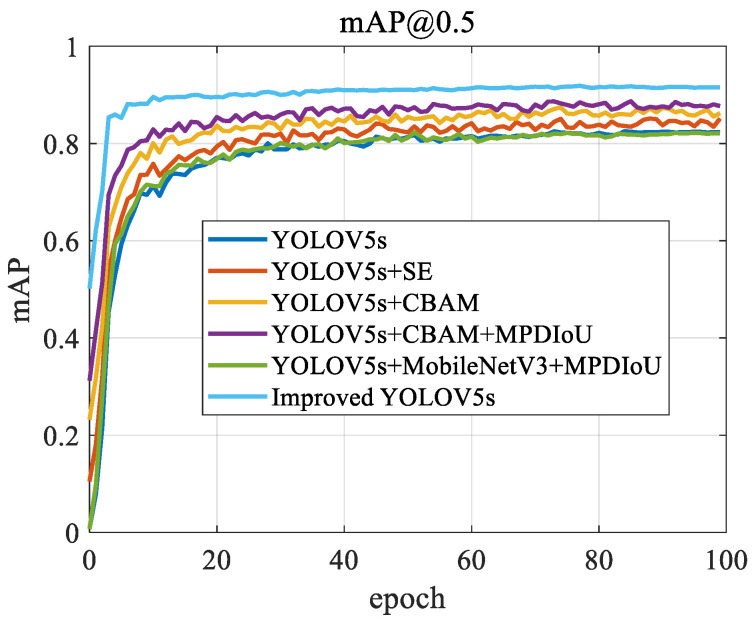
mAP@0.5 curve.

The mAP@0.5 curve throughout the model’s training process is shown Figure 7:

The improved YOLOv5s shows the most stable and highest mAP@0.5 values, consistently staying above 0.9 throughout the training process, demonstrating its superior detection accuracy and model stability. Other models, such as YOLOv5s+CBAM+MPDIoU and YOLOv5s+MobileNetV3+MPDIoU, also perform well, but their mAP is slightly lower than that of improved YOLOv5s. This may be due to the more effective feature extraction and optimization techniques used in improved YOLOv5s. The baseline YOLOv5s model shows rapid performance gains during the early stages of training, but its growth plateaus and eventually stabilizes at a lower mAP level, suggesting that the basic model may have limitations in handling complex or diverse scenarios. YOLOv5s+SE and YOLOv5s+CBAM show steady performance improvements, reflecting the contributions of SE and CBAM attention mechanisms in enhancing the model’s overall recognition ability, though they still do not reach the level of improved YOLOv5s.

**Figure 8:**
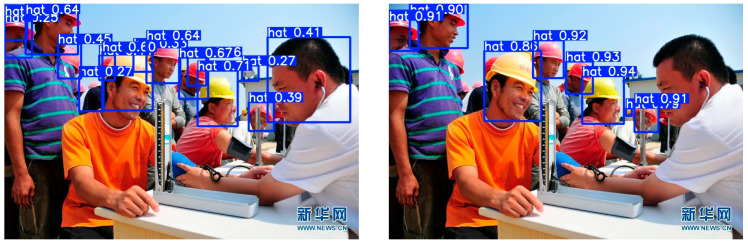
Comparison of basic YOLOV5s and improved YOLOV5s examples. (**Left**) Basic YOLOV5s; (**right**) improved YOLOV5s.

Figure 8 shows an example of an actual image. For the basic YOLOV5s and improved YOLOV5s algorithms, we can see from the results that although the basic YOLOV5s algorithm can locate the position of the helmet for the most part, the overall recognition confidence is low, and there is even a misjudgment of identifying a person as a hat. The improved YOLOV5s algorithm can also locate the position of the helmet, and the confidence is generally high, and there is no misjudgment.

**Figure 9:**
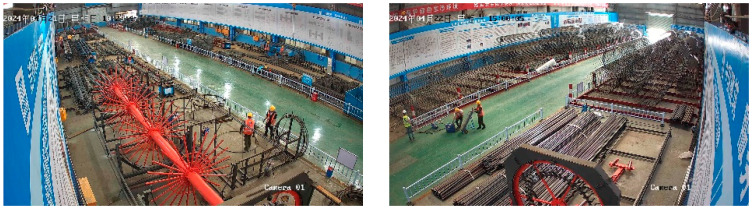
Images of the self-made small target dataset.

**Figure 10:**
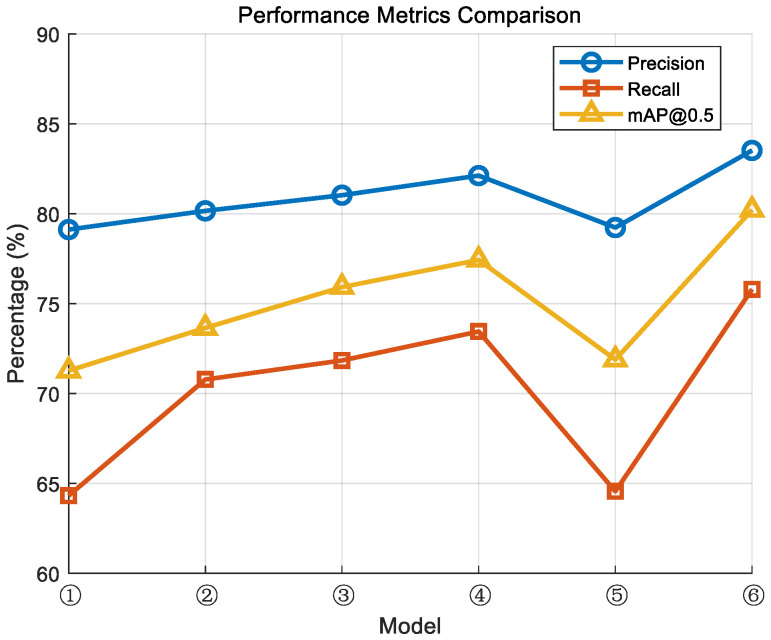
Self-made dataset training results.

In order to verify that this method can also detect good results on other datasets, we contacted China Construction Second Engineering Bureau Co., Ltd. (Beijing, China) to collect some pictures of the construction site. These pictures are all captured by cameras on the construction site, so the pixel proportion of workers in the camera is very small. Therefore, it can be considered that this dataset can be used as a small target detection dataset. The dataset picture is shown in Figure 9:

The model was trained using the method mentioned in this article, and the experimental results are as Figure 10:

From the above results, it is evident that different models exhibit a gradual improvement trend in precision, recall, and mAP@0.5 metrics. In particular, the improved model ⑥ achieved optimal performance across all three metrics, with a precision of 83.52%, a recall of 75.79%, and an mAP@0.5 of 80.21%. In contrast, the performance of model ① was relatively weaker, indicating that without improvements, YOLOv5s is less effective in detecting small objects.

It is particularly noteworthy that the recall metric exhibits notable fluctuations, especially in small object detection, where recall is significantly lower than precision. This suggests that the model faces challenges in localizing small objects, resulting in the failure to detect some of them. This is due to the fact that in the non-optimized model, small object information is easily lost during feature extraction, impacting the final detection accuracy.

**Figure 11:**
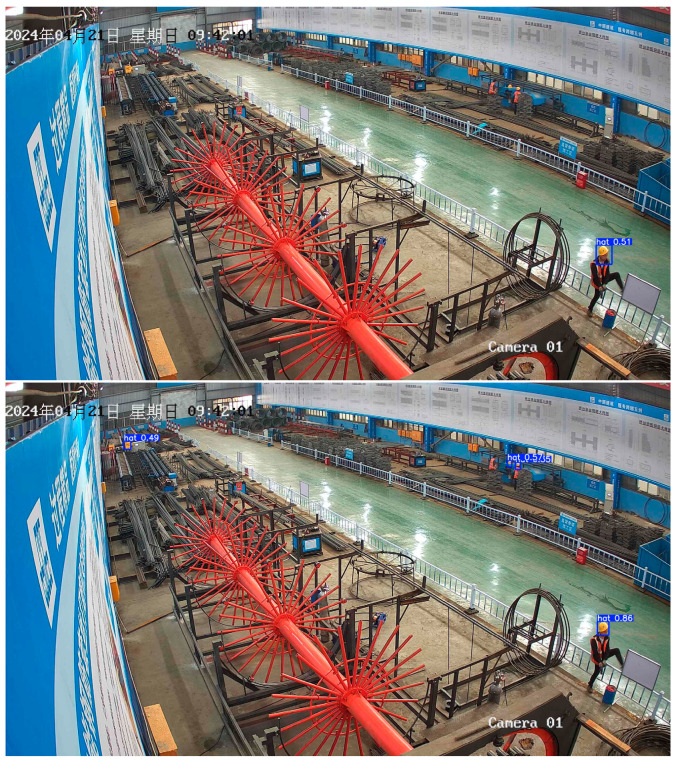
Small object detection images. (**upper**) Basic YOLOV5s; (**down**) improved YOLOV5s.

To resolve this issue, a small object detection layer was added to the improved algorithm, enhancing its ability to detect small objects. To visually demonstrate the differences in performance before and after the improvements, two comparative images will be shown: one illustrating the non-improved algorithm’s performance, and the other showing the improved algorithm under the same conditions. These images clearly highlight the enhanced effectiveness of the improved algorithm in detecting small objects. The result shown as Figure 11.

## 5. Conclusions

Based on the experimental analysis conducted in this study, the following key conclusions were drawn:

Improvement in Model Performance:

The improved YOLOv5s model, by integrating MobileNetV3, the CBAM attention mechanism, and the MPDIoU strategy, demonstrated excellent performance in both precision (93.81%) and recall (86.33%). This represents a significant improvement compared to the baseline model, which had a precision of 89.56% and a recall of 75.41%.

Optimization of Computational Efficiency:

By incorporating the lightweight MobileNetV3 structure, the FLOPs of the improved model were significantly reduced to 5.7G, indicating that the model effectively reduces the consumption of computing resources while maintaining high accuracy, making it suitable for resource-constrained real-world applications.

Reliability in Practical Applications:

The improved YOLOv5s model exhibited higher confidence and lower false positive rates in actual detection tasks. Especially in complex scenarios, it was able to more accurately identify and locate the position of safety helmets, demonstrating its stability and reliability in practical applications.

Advantages of Algorithm Integration:

The integration of various advanced algorithms (such as CBAM and MPDIoU) in this study significantly enhanced the robustness and adaptability of the object detection model, providing valuable practical experience for building efficient object detection systems.

## Figures and Tables

**Table 1:** Experimental environment.

Component	Model/Specifications
CPU	13th Intel Core i7-13700KF
RAM	32G
GPU	NVIDA GeForce RTX 4070 12 GB
Programing language	Python 3.8
Deep learning framework	PyTorch 2.3.1
CUDA	11.8

**Table 2:** Ablation experiment results comparison.

Model	Precision/%	Recall/%	mAP@0.5/%	FLOPs/G
ST-CenterNet [23]	94.19	71.88	89.06	-
YOLOv8s_ASPP_NWD [24]	91.8	86.6	92.0	-
①	89.56	75.41	82.34	15.8
②	90.47	81.51	85.12	15.8
③	91.24	82.38	86.23	15.8
④	92.44	84.30	87.70	15.8
⑤	89.90	75.24	82.06	5.7
⑥	93.81	86.33	91.56	5.7

## Data Availability

The original contributions presented in the study are included in the article, further inquiries can be directed to the corresponding authors.
